# Associations of Grandparenting Dimensions/Styles with Mental Health in Children and Adolescents: A Systematic Review and Meta-Analysis

**DOI:** 10.3390/bs15020180

**Published:** 2025-02-08

**Authors:** Lifen Zhao, Maoye Tian, Zhiyou Wang, Dandan Hu

**Affiliations:** 1 Department of Social Security, Nanjing Normal University, Nanjing 210024, China; lfzhao@njnu.edu.cn (L.Z.); 221702004@njnu.edu.cn (M.T.); 16220116@njnu.edu.cn (D.H.); 2 Department of Social Work and Social Policy, Nankai University, Tianjin 300350, China

**Keywords:** grandparenting, mental health, children, adolescent, systematic review, meta-analysis

## Abstract

In recent decades, grandparents’ increased involvement in child-rearing around the world has accelerated research on grandparenting. However, findings have remained inconsistent, and no reviews have examined how grandparenting dimensions and styles affect child and adolescent mental health. In our systematic review and meta-analysis, we synthesized evidence on the relationship between dimensions and styles of grandparenting and children’s and adolescents’ mental health. In nine databases, we identified 3197 studies on the relationship between dimensions and styles of grandparenting and children’s and adolescents’ mental health, 20 of which we ultimately reviewed. To better integrate the results, we performed a meta-analysis of studies addressing the same mental health outcomes (i.e., depression, anxiety, and internalizing symptoms). Moreover, to synthesize evidence from the studies, we conducted both random- and common-effects meta-analyses. The reviewed studies involved 11,434 children overall. Among our findings, the associations between positive dimensions and styles of grandparenting and children’s and adolescents’ depression and anxiety were moderately significant (depression: r = −0.33; anxiety: r = −0.12), as were the correlations between negative dimensions and styles and all three mental health outcomes (depression: r = 0.15; anxiety: r = 0.15; internalizing symptoms: r = 0.25). In general, positive dimensions and styles of grandparenting are negatively associated with mental health conditions, whereas negative ones are positively associated. In this article, we discuss those and other findings and propose directions for future studies.

## 1. Introduction

Childhood and adolescence can be volatile periods of rapid biological and psychosocial development characterized by significant fluctuations in depressive symptoms, anxiety, and related mental health conditions (Kazdin, 1990). Thus, for countries around the world, those mental health conditions among children and adolescents have become prominent challenges for social and public health (Lawrance, 2024). Recent research on the global burden of disease found that, in 2019, 280 million people suffered from depression—approximately 3.8% of the global population—including 23 million children and adolescents, while 301 million people suffered from anxiety disorders—approximately 4% of the global population—including 58 million children and adolescents (World Health Organization, 2023). According to the China National Mental Health Development Report (2021–2022), in 2020, the risk of depression was 11.4% among students in Grades 4–6 and 26.6% among students in Grades 7–9 (X. H. Jiang, 2019).

The family, as the first social environment in an individual’s life, is critical in understanding children’s and adolescents’ mental health conditions (Ani, 2024). As modern societies experience changes in family structures and functions, the involvement of grandparents in childrearing has increased worldwide (Buchanan & Rotkirch, 2018; J.-P. Tan et al., 2010). Unlike grandparents who only provide financial support and occasionally visit their grandchildren, grandparents who raise grandchildren have two distinct characteristics: (1) they are usually the primary caregivers of grandchildren, assuming parental responsibilities that include day-to-day caregiving and discipline (Dolbin-MacNab & Yancura, 2018; Hayslip & Kaminski, 2005); (2) they face ongoing intense parenting stress, including financial burdens, psychological pressures, inability to solve problems, and lack of social support (Xu et al., 2020), which affect their grandparenting practices and, in turn, grandchildren’s mental health (Smith & Palmieri, 2007). This study focuses on grandparents raising grandchildren.

According to the U.S. Census Bureau, in 2021, 32.7% of grandparents were responsible for the care of grandchildren under the age of 18, and about one–third cared for children under the age of 6 (U.S. Census Bureau, 2024). In China, data from a survey by the Chinese Centre for the Aging shows that 60% to 70% of children aged 0 to 2 years nationwide are primarily cared for by grandparents (National Health and Family Planning Commission, 2015). In Western countries, grandparents typically become the primary caregivers and assume the role of daily care and guardianship when parents are unable to provide care due to incarceration, substance abuse, or death (Hadfield, 2014; Mutchler & Baker, 2004; Sadruddin et al., 2019). By contrast, grandparental care is seen as “culturally normative and expected” in Asian societies, which are characterized by collectivist and familism culture (Burnette et al., 2013), aiming at helping the working middle generation and improving the well-being of the family as a whole (C. D. Wang et al., 2019a).

As grandparents increasingly assume the role of primary caregivers for children and adolescents worldwide, the impact of grandparental care on child development has received considerable attention. Numerous studies have begun to compare the developmental outcomes of grandchildren cared for by their grandparents with those of grandchildren raised by parents (Edwards, 2009; Kelley et al., 2011). These studies have identified significant mental and general health problems among grandchildren in grandparent care, such as increased emotional difficulties and externalizing problems (Edwards, 2006; Kelley et al., 2011). In addition, reviews synthesizing multiple studies have reached similar conclusions (Sadruddin et al., 2019; Y. Wang et al., 2024; Xu et al., 2022), demonstrating that children in grandparental care have higher levels of internalizing problems and overall mental health difficulties compared to their peers. However, while these reviews have broadly examined the general impact of grandparental care (yes or no) on child and adolescent development, relatively few have focused on the impact of specific dimensions and styles of grandparenting on child and adolescent mental health. Given the increasing prevalence of grandparent caregiving worldwide, it is particularly important to systematically explore how dimensions and styles of grandparenting styles influence children’s and adolescents’ mental health.

### 1.1. Dimensions and Styles of Grandparenting

Since both parents and grandparents are important caregivers and sources of social agency in the lives of children and adolescents, dimensions and styles of grandparenting in existing research have been largely been adapted from dimensions of parenting (Kelley et al., 2011; Y. Wang et al., 2024).

In the literature on parenting, two approaches for operationalizing parenting have been adopted: a dimensional approach focusing on individual dimensions of parental behaviors, including parental warmth and negligence, and a categorical approach that categorizes parenting behaviors into parenting styles according to a combination of those dimensions (Maccoby and Martin, 1983). At the same time, the literature presents varying operationalizations of parenting. Skinner et al. (2005) have classified parenting into positive parenting and negative parenting; positive parenting includes the dimensions of parental warmth (i.e., emotional support and expressions of affection), support with autonomy (i.e., encouragement of independence, decision-making, and self-expression), and structure (i.e., clear boundaries and consistent interactions), whereas negative parenting includes the dimensions of parental rejection (i.e., dislike, hostility, and neglect), coercion (i.e., force, threats, and manipulation designed to control), and chaos (i.e., inconsistent parenting, disorganization, and unpredictability) (Skinner et al., 2005). Regarding the second approach, parenting styles are formed by combinations of dimensions of parenting. Maccoby and Martin (1983), using a framework of responsiveness and warmth versus demandingness and control, have categorized parenting into four styles: authoritative style, characterized by high levels of warmth and control; authoritarian style, marked by a low level of warmth and a high level of control; permissive style, characterized by a high level of warmth and limited control; and neglectful style, defined by low levels of both warmth and control.

As previously noted, the dimensions of grandparenting are largely derived from those of parenting. Similar to parenting, the dimensions and styles of grandparenting have shown inconsistency across studies to date. G. Y. Liu et al. (2023) have categorized grandparenting into two dimensions—nurturing and psychological pressure—whereas Yang and Liu (Yang & Liu, 2020) have delineated three dimensions: rejection, emotional warmth, and overprotection. Other authors have classified grandparenting styles according to positive and negative approaches; positive grandparenting encompasses warmth, supervision, and reasonable explanations, whereas negative grandparenting involves hostility, inconsistent parenting attitudes, and harshness (P. Li & Li, 2022). Moreover, Wei (2018), who conducted a cluster analysis on the three dimensions of attentive parenting, concise parenting, and supportive parenting, has identified four grandparenting styles: intermediate, neglectful, warm, and intrusive.

### 1.2. Dimensions and Styles of Grandparenting and Mental Health

Current research has predominantly focused on comparing the development of children cared for by their grandparents with those of children raised by parents (Edwards, 2009; Kelley et al., 2011), with less attention paid to the effect of specific dimensions and styles of grandparenting. A few studies explored the relationship between dimensions and styles of grandparenting and depression, and have indicated that positive grandparenting practices (e.g., warmth) negatively influence children’s depression, whereas negative ones (e.g., psychological control) significantly exacerbate depressive symptoms (P. Li & Li, 2022; Y. Tan et al., 2023). However, other studies have shown conflicting conclusions. For instance, Kong (2010) found no correlation between positive grandparenting (i.e., nurturance) and children’s depression. Meanwhile, Keller et al. (2023) have suggested that positive grandparenting practices can significantly mitigate children’s anxiety and stress, whereas dysfunctional grandparenting is associated with a marked increase in internalizing symptoms (Smith et al., 2008).

### 1.3. Current Study

Existing reviews have mainly focused on the effect of grandparental care (yes or no) on children’s physical health (Pulgaron et al., 2016), (over)weight (An et al., 2020), educational outcomes (Anderson et al., 2018), social and behavioral development (Sadruddin et al., 2019), and mental health (Y. Wang et al., 2024; Xu et al., 2022). Most reviews have consistently concluded that children raised by their grandparents versus their parents have poorer development, usually with more internalizing problems, overall mental health issues (Y. Wang et al., 2024), and behavioral problems (Xu et al., 2022). By contrast, relatively few reviews have focused on the specific dimensions and styles of grandparenting and children’s and adolescents’ development, let alone how dimensions and styles of grandparenting affect their mental health. Even so, many reviews have focused on the relationship between dimensions and styles of parenting and children’s mental health (X. L. Zhang et al., 2022). For example, one review from Portugal has also demonstrated that an authoritative parenting style can prevent children’s internalizing and externalizing problems (Pulquério de Paula, 2012). In sum, reviews on how dimensions and styles of grandparenting relate to children’s and adolescents’ mental health have been sorely limited, and the topic demands additional attention.

Although past empirical studies, systematic reviews, and meta-analyses have provided important references for the relationship between dimensions and styles of grandparenting and children’s and adolescents’ mental health, two gaps remain. On the one hand, mixed effects have been found in the relationship between dimensions and styles of grandparenting and children’s and adolescents’ mental health. On the other, at least to our knowledge, no systematic reviews or meta-analyses have been conducted on the effects of dimensions and styles of grandparenting on children’s and adolescents’ mental health. Thus, the research question of the present study is raised: What are the associations between dimensions and styles of grandparenting and children’s and adolescents’ mental health? In our systematic review, we aimed to synthesize the findings of empirical studies explore the effect of dimensions and styles of grandparenting on children’s and adolescents’ mental health.

## 2. Methods

### 2.1. Literature Search

To retrieve as many relevant studies as possible, we comprehensively searched the Chinese and English literature from January 2000 to August 2024 for studies on dimensions and styles of grandparenting in relation to children’s and adolescents’ mental health. To that end, we searched nine databases, including five English ones (i.e., Springer Link, Web of Science, Elsevier SD, EBSCO, and PubMed) and four Chinese ones: China National Knowledge Infrastructure (知网), Wanfang Data Knowledge Service Platform (万方), CQVIP (维普), and China Masters’ Theses Full-Text Database (中国优秀硕士学位论文全文数据库). Searches were conducted on titles and abstracts using terms related to dimensions and styles of grandparenting (i.e., “grandparenting” OR “grandparenting styles” OR “reared by grandparents” OR “grandparent parenting style” OR “grandparent parenting style” OR “grandparent education”) and mental health (i.e., “mental health” OR “mental illness” OR “disorder” OR “depression” OR “anxiety” OR “internalizing problem”). Translated search terms were used to identify the relevant Chinese literature, and the Boolean operator “AND” combined the two search components.

### 2.2. Selection Criteria

Following the PICoS principle, which prioritizes the population, phenomena of interest, context, and study design of research, we adopted five inclusion criteria, such that studies had to (1) examine children or adolescents (i.e., <18 years old) as participants; (2) empirically examine dimensions and styles of grandparenting and mental health but not be reports or reviews; (3) report the correlation coefficient between those dimensions and styles and mental health or other indicators that could be converted into effect size; (4) report the sample size; and (5) be based on independent samples, meaning that if multiple studies used the same sample and measurements, then only one of the studies was included.

### 2.3. Data Extraction

**Figure 1:**
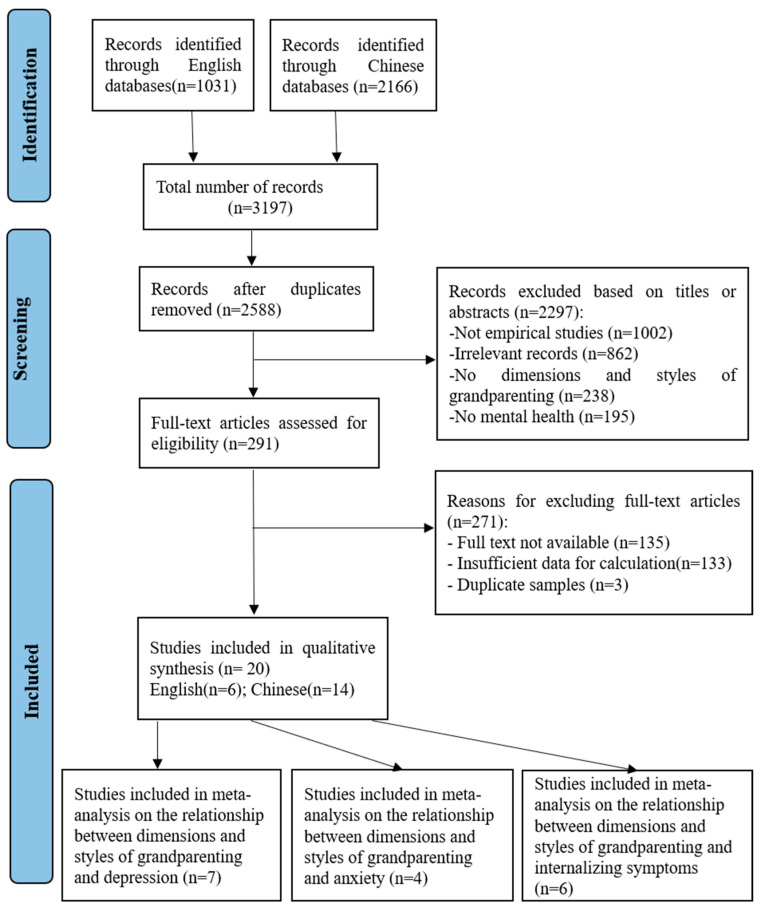
Illustration of search results and procedures for article selection.

All eligible literature extracted from the databases was imported into Endnote, and duplicate references were removed. The literature was screened according to the PRISMA guidelines. First, the studies were screened by title and abstract. Second, two authors read and evaluated the full text of the studies separately, and differences in the assessment were resolved through discussion with the third author. Third, all studies that met the inclusion criteria were identified. The specific flow chart for the literature search appears in Figure 1, while key information about the included studies, extracted and tabulated to aid comparison and synthesis, appears in Table 1.

### 2.4. Data Analysis

R statistical software 4.3.1 was used for data analysis. The Pearson correlation coefficient r was used as the effect size, and if that value was not provided, then data that could be converted into r values, including F, t, or χ^2^ values, were collected, converted to Fisher’s z values, and finally converted to r values (W. H. Jiang et al., 2011). In one meta-analysis on how parenting styles influence suicidal ideation among adolescents, Gao et al. (2022) divided parenting styles into positive and negative styles, such that each pathway from parenting to mental health produced an effect size. If any study examined multiple dimensions of grandparenting and mental health simultaneously, then they were coded separately to produce multiple effect sizes.

Cochran’s Q test, yielding a Q statistic, was employed to assess heterogeneity between studies. The larger the Q value, the smaller the corresponding *p* value. If *p* < α, then heterogeneity between studies was confirmed. The chi-square test was also used to identify any heterogeneity between studies. In the I^2^ test, 25%, 50%, and 75% indicated low, medium, and high heterogeneity, respectively. Based on the above, if no or low heterogeneity existed between the studies (*p* > 0.1, I^2^ < 50%), then we used a fixed-effects model for the combined analysis of data (Zhai and Guyatt, 2024; Zhan, 2017). If there was heterogeneity (*p* < 0.1, I^2^ > 50%), then a random effects model was used. For the interpretation of r, |r| ≤ 0.10 indicated a low correlation, 0.10 < |r| < 0.4 indicated a moderate correlation, and |r| ≥ 0.40 indicated a high correlation (Lipsey & Wilson, 2001).

## 3. Results

### 3.1. Search Outcomes and Study Characteristics

A total of 3197 studies were gathered during our search: 1031 in English and 2166 in Chinese. Of the 2588 studies remaining after duplicates were removed, a review of titles and abstracts revealed 2297 papers that did not meet the inclusion criteria, including unrelated studies and non-empirical studies, and they were excluded, leaving 291 full-text studies. Next, studies with incomplete data and duplicate samples and without correlation coefficients reported were excluded. Ultimately, 20 eligible studies were included in our review, all of which examined the relationship between dimensions and styles of grandparenting and depression (*n* = 7), anxiety (*n* = 4), or internalizing symptoms (*n* = 6).

Of the included studies, 18 were conducted in Chinese contexts, one was conducted in the United States (Keller et al., 2023), and another was conducted in Spain (Smith et al., 2008). Notably, grandparents take on caring responsibilities in East Asian countries mostly under the influence of collectivism and to support the middle generation of working adults (C. D. Wang et al., 2019a). By contrast, the reasons for grandparenting in Western societies included parental drug abuse, parental incarceration, and/or child abuse (Downie et al., 2010). Overall, 11,434 child and adolescent participants were involved in the 20 studies, with ages ranging from 3 to 18 years old and boys accounting for 45.01% to 59.20%. Only five studies reported the average age of grandparents, ranging from 56 to 67.65 (Keller et al., 2023; Kong, 2010; Kong et al., 2012; Smith et al., 2008; Wu, 2016). All included studies were cross-sectional. Most participants in the studies were recruited through schools (i.e., thirteen in primary and secondary schools and four in kindergartens), whereas participants in three studies were recruited from the community, social service organizations, or the Internet. In four studies, the dimensions and styles of grandparenting and children’s or adolescents’ mental health were reported by students (Gong, 2017; W. H. Jiang et al., 2011; X. Li, 2021; Y. Li et al., 2019; P. Li & Li, 2022; Peng, 2023; Yan, 2018); in four studies, caregivers reported their parenting styles but children or adolescents reported their mental health (Keller et al., 2023; Kong, 2010; Kong et al., 2012; Y. Tan et al., 2023); in five studies, caregivers reported their parenting styles and the children’s or adolescents’ mental health (Gao et al., 2022; Miao, 2015; Smith et al., 2008; L. F. Wang, 2007; Wu, 2016); and in one study, grandparenting was reported by grandparents, whereas children’s or adolescents’ mental health was reported by teachers (Xue, 2013). Eight studies reported the proportion of grandmothers. In two of these studies, the proportion of grandmothers was less than 50% (Kong, 2010; Kong et al., 2012), while in the remaining six studies, the proportion of grandmothers exceeded 75% (Peng, 2023; Smith et al., 2008; Y. Tan et al., 2023; Wu, 2016; Yan, 2018; W. Y. Zhang, 2021). Beyond that, three studies explored the effects of grandmothers’ and grandfathers’ parenting on children’s and adolescents’ mental health, respectively (Gong, 2017; Kong, 2010; W. Y. Zhang, 2021), while three studies focused exclusively on the effects of grandmothers’ parenting on mental health (Smith et al., 2008; Y. Tan et al., 2023; Yan, 2018). The remaining studies did not focus on the gender of the grandparents but only on the impact of dimensions and styles of grandparenting on the mental health of children and adolescents in general.

### 3.2. Data Extraction and Quality Assessment

A coding sheet was developed to define the variables in each study. The items were coded as follows: (a) first author and year of publication, (b) country, (c) sample size, (d) mean age of children and adolescents, (e) measurement of dimensions and styles of grandparenting, (f) measurement of mental health, (g) effect size, and (h) quality rating (Table 1).

We used the Combie scale, a cross-sectional study quality assessment tool, to assess the risk of bias and the quality of the included studies (Meng and Zhang, 2023). The scale includes seven indicators for evaluation, including scientific research design, reasonable data collection strategy, representative sample, and reasonable method. If the above criteria are met, then a score of 1 is assigned; if not, then a score of 0 is assigned; and if it is unclear whether they are met, then a score of 0.5 is assigned. The total score of the scale is 7.0 points, with 5.5–7.0 points conferring a grade of A, 4.0–5.5 points conferring a grade of B, and less than 4.0 points conferring a grade of C. Two authors assessed the quality of each study separately and gave an overall score of A, B, or C. If the results were inconsistent, then both authors held a discussion with the third author until consensus was reached. Overall, the average score of the included studies was 5.6 points, with 10 studies graded B and 10 studies graded A (Table 1).

### 3.3. Associations of Dimensions and Styles of Grandparenting with Mental Health

Studies to date have not consistently operationalized dimensions or styles of grandparenting. Yan (2018) and W. Y. Zhang (2021) have divided grandparenting into six dimensions: emotional warmth, harsh punishment, excessive interference, preference, rejection and denial, and overprotection. By comparison, Yang and Liu (2020) have posited that grandparenting includes three dimensions (i.e., rejection, warmth, and over-protection), while others have included only two dimensions: nurturance and psychological stress (Kong, 2010; Kong et al., 2012; Miao, 2015; Xue, 2013). To measure variables, five studies have used scales adapted from the Parent Behavior Report (Kong, 2010; Kong et al., 2012; G. Y. Liu et al., 2023; Miao, 2015; Xue, 2013), three from the Egna Minnen Beträffande Uppfostran (Peng, 2023; Yan, 2018; W. Y. Zhang, 2021), and two from Egna Minnen Beträffande Uppfostran (X. Li, 2021; Yang & Liu, 2020). All other studies employed scales compiled by the authors or entirely different scales—for instance, the Parenting Practices Interview used by Smith et al. (2008). Thus, there does not seem to be any consistent, widely used measurement tool for dimensions and styles of grandparenting.

Similarly, no consistency emerged in the measurement of mental health. For example, three studies used the Strengths and Difficulties Questionnaire to measure children’s internalizing problems (Gao et al., 2022; G. Y. Liu et al., 2023; Smith et al., 2008), while four chose the Child Behavioral Checklist and Children’s Depression Inventory to measure children’s depression (P. Li & Li, 2022; Keller et al., 2023; Kong et al., 2012; Yan, 2018). All other studies used scales such as the Loneliness and Social Dissatisfaction Scale and the Middle School Student Mental Health Scale to measure psychological conditions such as loneliness and anxiety (Kong, 2010; W. Y. Zhang, 2021).

Regarding the effect of dimensions and styles of grandparenting on mental health, the included studies all showed that such dimensions and styles promoted good mental health outcomes, whereas negative ones predicted relatively severe mental health conditions. In particular, grandparental warmth significantly reduced children’s depressive symptoms (Yang & Liu, 2020) and enhanced their resilience (X. Li, 2021). Other studies have also shown that rejection and/or neglect from grandparents can exacerbate children’s emotional troubles (Miao, 2015), while grandparents’ excessive interference, preference, and/or control can exacerbate their obsessive–compulsive, depressive (Yan, 2018), anxiety, and internalizing symptoms (Gao et al., 2022; W. Y. Zhang, 2021).

### 3.4. Theoretical Foundation

Of the 20 studies reviewed, eight (40%) were not guided by any theories. Of the studies that were, eight cited ecological systems theory (Gong, 2017; X. H. Jiang, 2019; Kong, 2010; Kong et al., 2012; X. Li, 2021; P. Li & Li, 2022; Miao, 2015; Yan, 2018), two cited family systems theory (Gao et al., 2022; Kong, 2010), one cited self-determination theory (Gao et al., 2022), one cited the family stress model (Smith et al., 2008), one cited crossover theory (Y. Tan et al., 2023), and one cited Erikson’s theory of psychosocial development (L. F. Wang, 2007). Altogether, ecological systems theory has been the most widely used theory in such research.

Ecological systems theory emphasizes the importance of individual characteristics and social environment in individuals’ development. Grandparents are important members of the child’s microsystem, and their parenting styles and interactions with other members of the system affect the child’s development (Bronfenbrenner, 1979). On that count, P. Li and Li (2022) found that left-behind children with absent parents were more likely than other children to be subjected to negative grandparenting styles, which led to severe depressive symptoms. From the perspective of ecological systems theory, such disruptions within the microsystem, particularly the negative interactions between grandparents and children, contribute to poorer mental health outcomes in left-behind children. Meanwhile, Gong (2017) showed that grandparents’ authoritative parenting styles negatively predicted children’s anxiety, whereas permissive and authoritarian parenting styles exacerbated grandchildren’s behavioral problems. According to ecological systems theory, an authoritative grandparenting style fosters a stable and nurturing microsystem, which supports children’s emotional development. In contrast, permissive and authoritarian styles disrupt the balance of the microsystem, thereby adversely affecting children’s behavioral outcomes. According to Miao (2015), the family environment is an important microsystem that influences children’s psychological development, and negative grandparenting styles significantly and positively predict children’s social withdrawal, anxiety, and depression. However, many studies have merely mentioned the theory, and the theory has not heavily guided the research, including in measuring concepts, making assumptions about the relationship between variables, and developing theoretical models.

Similar to ecological systems theory, the family stress model and Erikson’s theory of psychosocial development have been employed to emphasize the role of dimensions and styles of grandparenting in children’s and adolescents’ mental health, whereas family system theory and crossover theory have been applied to provide theoretical foundations to examine the interactive effects of parental and grandparental psychological control on children’s development in multigenerational families. For example, guided by crossover theory, Y. Tan et al. (2023) found that grandmothers’ psychological and behavioral control were indirectly associated with children’s depressive symptoms through mother–child attachment. This finding suggests that grandparental and parental psychological control interact and spill over, collectively influencing children’s development. By contrast, although self-determination theory sheds light on the mechanism of grandparenting in relation to mental health, because such psychological control thwarts one’s universal psychological needs and leads to diverse maladjustments (Vansteenkiste et al., 2020), the mechanism was not tested by Gao et al. (2022).

### 3.5. Quantitative Synthesis

As mental health outcome measures varied across the studies, we were unable to perform a meta-analysis on all studies. Instead, those focused on the same mental health outcomes (i.e., depression, anxiety, internalizing symptoms) were integrated. For the sake of brevity, only significant results are displayed.

#### 3.5.1. Dimensions and Styles of Grandparenting and Depression

Seven studies were included in our meta-analysis of the relationship between dimensions and styles of grandparenting and depression. Among them, six were included in the analysis of the association between positive dimensions and styles and depression (Keller et al., 2023; Kong, 2010; P. Li & Li, 2022; Y. Tan et al., 2023; Wei, 2018; Yan, 2018; Yang & Liu, 2020; W. Y. Zhang, 2021), and six of the same or other studies were included in the analysis of the relationship between depression and negative dimensions and styles (Keller et al., 2023; Kong, 2010; P. Li & Li, 2022; Y. Tan et al., 2023; Wei, 2018; Yan, 2018; Yang & Liu, 2020; W. Y. Zhang, 2021).

**Figure 2:**
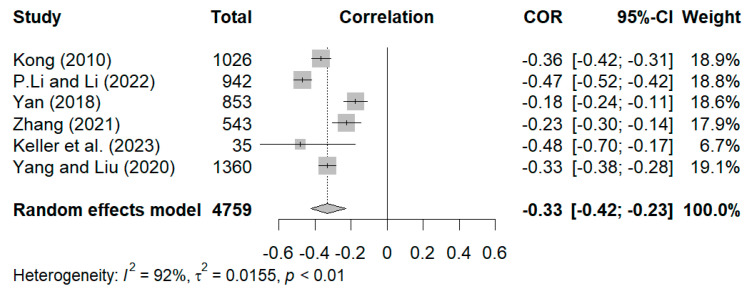
Forest plot of correlation between positive dimensions and styles of grandparenting and depression (Kong, 2010; P. Li & Li, 2022; Yan, 2018; W. Y. Zhang, 2021; Keller et al., 2023; Yang & Liu, 2020).

As shown in Figure 2, the overall random effect pooled estimate for the relationship between positive dimensions and styles of grandparenting and depression was r = −0.33. The 95% confidence interval (CI) was −0.42 to −0.23 (*p* < 0.01), which suggests a statistically significant relationship between positive dimensions and styles of grandparenting and depression. Despite the presence of substantial heterogeneity in the effect sizes of studies, due to a limited number of eligible studies we could not further examine the source of heterogeneity using subgroup analysis. Instead, we performed sensitivity analysis to investigate the robustness of the results. To begin, the study conducted by Yang and Liu (2020), which had a large weight, was excluded. After its exclusion, the results were still statistically significant (r = −0.33, 95% CI [−0.45, −0.20]). Afterward, the remaining studies were eliminated one by one, and their effect sizes, ranging from −0.36 to −0.29, were close to the total effect size of −0.33, thereby indicating that the results of meta-analysis were stable.

**Figure 3:**
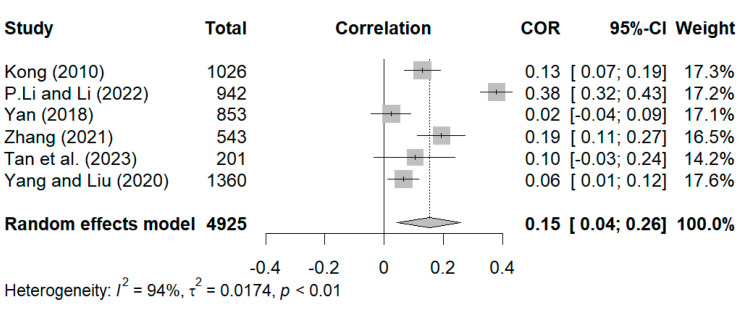
Forest plot of correlation between negative dimensions and styles of grandparenting and depression (Kong, 2010; P. Li & Li, 2022; Yan, 2018; W. Y. Zhang, 2021; Y. Tan et al., 2023; Yang & Liu, 2020).

For the relationship between depression and negative dimensions and styles of grandparenting, the overall random effect pooled estimate was r = 0.15. The 95% CI was 0.04 to 0.26 (*p* < 0.01), which suggests a statistically significant relationship between depression and negative dimensions and styles (Figure 3). For the sensitivity analysis, the study conducted by Yang and Liu (2020), which had a large weight, was excluded. After its exclusion, the results remained statistically significant (r = 0.17, 95% CI [0.05, 0.29]). Thereafter, the remaining studies were eliminated one by one, and their effect sizes (0.10–0.18) were close to the total effect size of 0.15, indicating that the results of the meta-analysis were stable.

#### 3.5.2. Dimensions and Styles of Grandparenting and Anxiety

Four studies were included in the statistical analysis of the relationship between dimensions and styles of grandparenting and anxiety. Among them, three involved both positive and negative dimensions and styles (Gong, 2017; Peng, 2023; W. Y. Zhang, 2021), whereas one only studied positive dimensions and styles (Keller et al., 2023).

**Figure 4:**
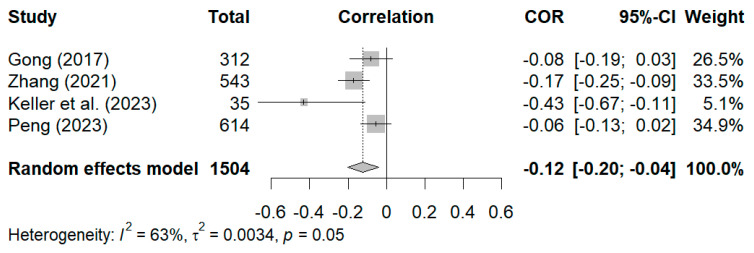
Forest plot of correlation between positive dimensions and styles of grandparenting and anxiety (Gong, 2017; W. Y. Zhang, 2021; Keller et al., 2023; Peng, 2023).

Four studies were included in the statistical analysis of the relationship between positive dimensions and styles of grandparenting and anxiety (Gong, 2017; Keller et al., 2023; Peng, 2023; W. Y. Zhang, 2021). As shown in Figure 4, the overall random effect pooled estimate was r = −0.12. The 95% CI was −0.20 to −0.04 (*p* < 0.01), which suggests a statistically significant relationship between positive dimensions and styles of grandparenting and anxiety. For the sensitivity analysis, Peng’s (2023) study, which had a large weight, was excluded. After its exclusion, the results remained statistically significant (r = −0.16, 95% CI [−0.26, −0.05]). Thereafter, the remaining studies were eliminated one by one, and their effect sizes, ranging from −0.16 to −0.08, were close to the total effect size of −0.12, thus indicating that the results of the meta-analysis were stable.

**Figure 5:**
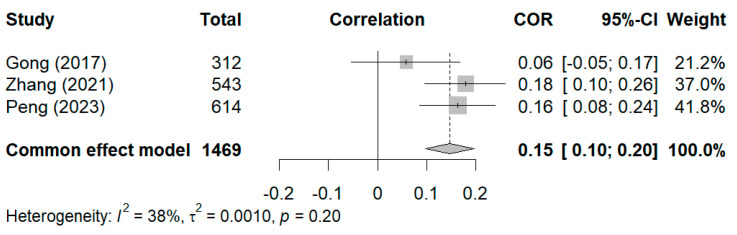
Forest plot of correlation between negative dimensions and styles of grandparenting and anxiety (Gong, 2017; W. Y. Zhang, 2021; Peng, 2023).

For the relationship between negative dimensions and styles of grandparenting and anxiety, a fixed-effects model was chosen because the heterogeneity test yielded a result of I^2^ < 50% (Zeng et al., 2024). As shown in Figure 5, the overall fixed effect pooled estimate was r = 0.15, and the 95% CI was 0.10 to 0.20 (*p* < 0.01), which suggests a statistically significant relationship between anxiety and negative dimensions and styles of grandparenting. For the sensitivity analysis, Peng’s (2023) study, which had a large weight, was also excluded. After its exclusion, the results remained statistically significant (r = 0.12, 95% CI [0.01, 0.24]). Thereafter, the remaining studies were excluded one by one, and their effect sizes (0.12–0.17) were close to the total effect size of 0.15, thereby indicating that the results were stable.

#### 3.5.3. Dimensions and Styles of Grandparenting and Internalizing Symptoms

Six studies were included in the statistical analysis of the relationship between dimensions and styles of grandparenting and internalizing symptoms. Among them, four examined both positive and negative effects of dimensions and styles of grandparenting on internalizing symptoms (Kong et al., 2012; Y. Li et al., 2019; G. Y. Liu et al., 2023; Wu, 2016), whereas two examined only the negative dimensions and styles (Gao et al., 2022; Smith et al., 2008).

**Figure 6:**
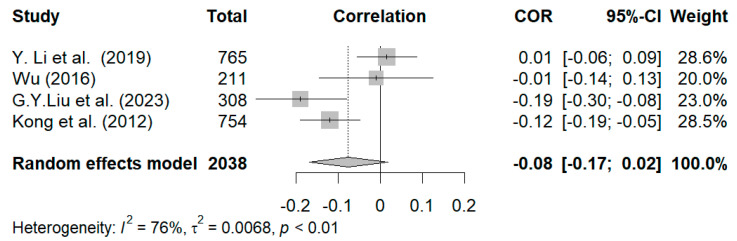
Forest plot of correlation between positive dimensions and styles of grandparenting and internalizing symptoms (Y. Li et al., 2019; Wu, 2016; G. Y. Liu et al., 2023; Kong et al., 2012).

Four studies were included in the statistical analysis of the relationship between positive grandparenting styles and internalizing symptoms (Kong et al., 2012; Y. Li et al., 2019; G. Y. Liu et al., 2023; Wu, 2016). The results of the random-effects model revealed that the effect size was relatively small (r = −0.08, 95% CI [−0.18, 0.02]) and not significant, as shown in Figure 6. Owing to the presence of heterogeneity in the study’s effect size and a limited number of eligible studies, sensitivity analysis was conducted to investigate the robustness of the results. Compared with the average age of participants in the other three studies (i.e., 14 months, 9.89 years, and 9.19 years, respectively), the participants in Y. Li et al.’s (2019) study were older, with an average age of 12.02 years, and mostly in Grades 4–6. To determine whether that result had a disproportionate effect on the summary effect size, the analysis was conducted again after removing Y. Li et al.’s study. As a result, the positive dimensions and styles of grandparenting were significantly related to internalizing symptoms, albeit moderately (r = −0.12, 95% CI [−0.21, −0.03], *p* < 0.01), thereby indicating that positive dimensions and styles in reducing internalizing symptoms among older children may not be as beneficial as for younger children.

**Figure 7:**
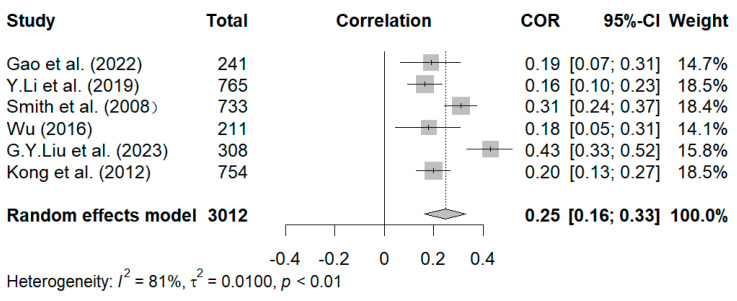
Forest plot of correlation between negative dimensions and styles of grandparenting and internalizing symptoms (Gao et al., 2022; Y. Li et al., 2019; Smith et al., 2008; Wu, 2016; G. Y. Liu et al., 2023; Kong et al., 2012).

Last, six studies measured the relationship between negative dimensions and styles of grandparenting and internalizing symptoms (Gao et al., 2022; Kong et al., 2012; Y. Li et al., 2019; Smith et al., 2008; Wu, 2016). As shown in Figure 7, the overall random effect pooled estimate was r = 0.25, and the 95% CI was 0.16 to 0.33 (*p* < 0.01), which suggests a statistically significant relationship between negative dimensions and styles of grandparenting and internalizing symptoms. For the sensitivity analysis, the research conducted by Y. Li et al. (2019), given its large weight, was excluded. After its exclusion, the results remained statistically significant (r = 0.27, 95% CI [0.17, 0.36]). Thereafter, the remaining studies were eliminated one by one, and the effect sizes (0.21–0.27) were close to the total effect size of 0.25, thereby indicating that the result was stable.

## 4. Discussion

In our systematic review, we examined the relationship between dimensions and styles of grandparenting and children’s and adolescents’ mental health. Overall, the meta-analysis showed that positive dimensions and styles of grandparenting were significantly and negatively associated with depression and anxiety in children and adolescents, whereas negative ones were positively associated with all three mental health outcomes (i.e., depression, anxiety, and internalizing symptoms). Although a few studies reported the significant effect of positive dimensions and styles of grandparenting on internalizing symptoms among children (G. Y. Liu et al., 2023; Kong et al., 2012), no overall effect was found in our synthesis of the studies.

The results correspond to the findings of previous systematic reviews and meta-analyses examining how dimensions and styles of parenting affect children’s mental health (Gorostiaga et al., 2019; Y. Liu & Merritt, 2018). Such correspondence suggests that negative parenting (e.g., harsh control, authoritarian parenting, and neglectful parenting) is associated with higher levels of internalizing symptoms, whereas positive parenting (e.g., parental warmth, support with autonomy, and authoritative parenting) is negatively associated with such symptoms (Pinquart, 2017). Although studies have largely focused on the role of parents and dimensions and styles of parenting—that is, not grandparents and grandparenting—they still provide critical references for our findings because both parents and grandparents are important caregivers and sources of social agency in children’s and adolescents’ lives. Caregivers’ warmth toward children and adolescents indicates that they are loved and appreciated, which can promote positive feelings and reduce the negative feelings of children and adolescents (Alegre et al., 2014). By contrast, caregivers’ psychologically controlling (e.g., shaming) behaviors may increase negative mental health outcomes among children and adolescents if they do not behave according to parental or grandparental expectations (Barber, 2002).

The chief focuses of studies thus far have been depression, anxiety, and internalizing symptoms. Our meta-analysis revealed that the effect size of positive dimensions and styles of grandparenting in relation to depression is larger (r = −0.33) than in relation to anxiety (r = −0.12). Those results align with the findings of a meta-analysis on parenting and children’s mental health by Yap et al. (2014), who found that parental warmth exerted a greater effect on depression (r = −0.29) than on anxiety (r = −0.15) in a series of cross-sectional studies. To our surprise, our meta-analysis also showed that positive dimensions and styles of grandparenting had no significant effect on internalizing symptoms. However, after excluding one study with older children (i.e., 12 years old on average), positive dimensions and styles of grandparenting were significantly correlated with internalizing symptoms, albeit only moderately. The results of our sensitivity analysis indicated that positive grandparenting’s impact on mental health is less for older children than for younger ones, possibly because most studies that we reviewed were conducted in Chinese contexts, where grandparental involvement tends to focus more on daily care than education (Sun, 2021). As children grow older and become increasingly independent, the role of grandparental care in the development of older children diminishes.

Furthermore, compared with the effect size of parental control (i.e., a kind of negative parenting) on children’s anxiety in a previous meta-analysis (r = 0.28) (Van Der Bruggen et al., 2008), the correlation of negative grandparenting with anxiety revealed by our meta-analysis was smaller (r = 0.15). The inconsistency may be due to two reasons. First, in their meta-analysis, Van der Bruggen et al. (2008) examined only the impact of parental control on children’s anxiety, whereas the negative dimensions and styles of grandparenting in our study included multiple dimensions (e.g., parental control, overprotection, and rejection). Other dimensions of negative grandparenting might have smaller effect sizes on children’s anxiety and thus may have resulted in a smaller combined effect size than in Van der Bruggen et al.’s study. Second, most studies included in our meta-analysis of negative grandparenting’s impact on children’s anxiety were conducted in Chinese contexts, which are generally characterized by collectivism and familism. Therein, grandparents often assist with child-rearing in multigenerational households and act as joint or secondary caregivers alongside the working parents (Lo and Lindsay, 2022). Therefore, the impact of grandparenting on children’s anxiety may be smaller.

According to family systems theory, the roles of all family members are interdependent, which underscores the potential joint influences of grandparenting and parenting and the relationship between caregivers and children on their development (J. Wang et al., 2019b). Several reviewed studies conducted in Chinese contexts have revealed the mediating roles of maternal parenting style (X. H. Jiang, 2019), parental care (Peng, 2023; W. Y. Zhang, 2021), mother–child attachment, and grandmother–child attachment (Kong, 2010) in the association between grandparenting and children’ mental health. Other mediators of the relationship between grandparenting and mental health include resilience (G. Y. Liu et al., 2023) and food insecurity (Yang & Liu, 2020). Because multigenerational families and grandparent–mother co-parenting are prevalent in Chinese culture, additional studies in such social contexts are needed to examine the potential interactive effects of parenting and grandparenting on children’s and adolescents’ mental health outcomes in multigenerational families. By comparison, in Western contexts, grandparents often assume responsibility for caring for children due to parental death, parental incarceration, and/or child abuse (Hayslip et al., 2019) and are thus the primary caregivers for children in the absence of parents. However, in such families where grandparents are the primary caregivers and parents are absent, the mechanisms through which dimensions and styles of grandparenting affect children’s and adolescents’ mental health remain unclear and warrant greater attention in future research.

## 5. Limitations

The findings of our study should be interpreted with caution due to several limitations. First, only studies in English and Chinese were reviewed, and except for one study from the United States and one from Spain, all were conducted in Chinese contexts. The lack of diversity in regions and languages may have contributed to the high heterogeneity and limited generalizability of the results. Second, all included studies were cross-sectional, which precluded drawing causal inferences regarding the relationship between dimensions and styles of grandparenting and mental health. While grandparenting is often considered to be a predictor of children’s and adolescents’ development, it is important to recognize that children’s and adolescents’ mental health outcomes can also impact the dimensions and styles of grandparenting. Third, because fewer than 10 studies were included in the meta-analysis, we could not assess publication bias using a funnel plot or Egger’s test, because having so few studies makes the power of the tests too low to distinguish chance from true asymmetry (Cochrane, 2024). Fourth and finally, notable variation emerged in the measurement of dimensions and styles of grandparenting and mental health across studies, with some studies utilizing scales developed by their authors that were not widely recognized, which limited their comparability.

## 6. Directions for Future Research

Because grandparents are increasingly the primary caregivers of children and adolescents around the world, the impact of grandparental care on child development has received significant attention in the past two decades. However, research on the specific role of dimensions and styles of grandparenting in children’s and adolescents’ mental health is only just beginning, and future research should further explore the topic.

For one, because the cross-sectional design of all studies included in our review precluded the evaluation of the causal association between dimensions and styles of grandparenting and mental health, longitudinal studies are needed to overcome that limitation. Future research should also develop high-quality, widely recognized measures for assessing dimensions and styles of grandparenting to facilitate comparability and/or synthesis across studies.

For another, according to kinkeeping theory, grandchildren are more likely to be closer to and value their relationships with grandparents who share their gender (Dubas, 2001). This is because grandmothers engage in activities such as cooking and storytelling with granddaughters, while grandfathers engage in similar masculine activities with grandsons. This leads to grandchildren being closer to and valuing same-gender grandparents due to shared experiences and cultural norms of gender roles (King & Elder, 1995; Troll et al., 1979). However, few studies have reported grandparents’ gender. Future research should thus further explore the gendered effects of dimensions and styles of grandparenting. In addition, caring for young children can be particularly challenging for older adults, who often have limited energy reserves (Hughes et al., 2007). Therefore, future research should include information on the age of grandparents to examine how age influences grandparenting styles and, in turn, the mental health of children and adolescents.

Beyond that, in the studies that we reviewed, 40% (*n* = 8) did not mention any theory. Of the 12 studies that did specify a theory, eight cited ecological systems theory. However, many researchers have failed to elaborate how the theory has been applied in their studies. In the future, researchers thus need to further develop the use of theory to guide the development of conceptual frameworks and carefully select theories that specifically emphasize the interrelationships between environmental systems and children in order to better explain the mechanisms that underpin the relationship between dimensions and styles of grandparenting and mental health.

Last, only two of the studies that we reviewed were from Western contexts. To date, Western research has focused on comparing the mental health of children and adolescents cared for by their grandparents with that of their counterparts, without probing the influence of dimensions and styles of grandparenting on mental health. For that reason, more research on the topic should be conducted in Western countries, which would facilitate comparisons with studies from Asian contexts and, in turn, deepen understandings of influence of dimensions and styles of grandparenting on children’ and adolescents’ mental health.

## Figures and Tables

**Table 1:** Summary of studies.

Author/Year	Country/ Region	Sample Size	Children’s Age	Grandparents’ Age	Proportion of Grandmothers	Measure/Scales		R		Quality
						Dimensions and Styles of Grandparenting	Mental Health	Positive	Negative	
1. Gao et al. (2022)	China	241	4.88	NA	NA	Psychological control/Psychological Control Questionnaire	Internalizing problem/Strengths and Difficulties Questionnaire (SDQ)	NA	0.190	A
2. Gong (2017)	China	312	11.35	NA	NA	Grandparenting styles/Adapted from parenting style questionnaire	Anxiety/Parent Symptom Questionnaire (PSQ)	−0.080	0.058	B
3. X. H. Jiang (2019)	China	762	NA	NA	NA	Grandparenting dimensions/Adapted from parenting style questionnaire	Well-being/Happiness Index Scale	0.270	−0.157	B
4. Kong (2010)	China	1026	8.38	66.41	41.91%	Grandparenting dimensions/Adapted from the Parent Behavior Report (PBR)	Loneliness/Loneliness and Social Dissatisfaction Scale (LSDS)	−0.115	0.120	B
							Depression/Children’s Depression Inventory (CDI)	−0.365	0.130	
							Social anxiety/Social Anxiety Scale for Children (SASC)	−0.030	0.230	
5. Kong et al. (2012)	China	754	9.19	65.59	46.32%	Grandparenting dimensions/Adapted from PBR	Internalizing Problem/Child Behavioral Checklist (CBCL)	−0.120	0.200	B
6. Keller et al. (2023)	Appalachian	35	12.56	67.65	NA	Grandparent positive parenting/Acceptance subscale of the Child Report of Parental Behavior Inventory (PBI)	Anxiety/Penn State Worry Questionnaire (PSWQ)	−0.430	NA	A
							Depression/Center for Epidemiologic Studies Depression scale (CESD)	−0.480	NA	
7. P. Li and Li (2022)	China	942	14.82	NA	NA	Grandparenting dimensions/Self-assessment parenting questionnaire from a youth and family programs	Depression/Child Depression Scale (CDI)	–0.470	0.380	B
8. X. Li (2021)	China	617	NA	NA	NA	Grandparenting dimensions/short form of the Egna Minnen Beträffande Uppfostran (s-EMBU)	Resilience/Brief Resilience Scale (BRS)	0.260	–0.080	B
9. Y. Li et al. (2019)	China	765	12.02	NA	NA	Grandparenting dimensions/Parental Bonding Instrument (PBI)	Internalizing problem/Youth Self Report (YSR)	0.015	0.165	A
10. G. Y. Liu et al. (2023)	China	710	9.89	NA	NA	Grandparenting dimensions/Adapted from PBR	Internalizing problem/Strengths and Difficulties Questionnaire (SDQ)	–0.190	0.430	A
11. Miao (2015)	China	269	NA	NA	NA	Grandparenting dimensions/Adapted from PBR	Anxiety and Depression/Child Behavior Checklist	0.030	0.150	A
12. Peng (2023)	China	614	NA	NA	91.37%	Grandparenting dimensions/Egna Minnen Beträffande Uppfostran (EMBU)	Anxiety/Mental Health Test (MHT)	–0.056	0.163	A
13. Smith et al. (2008)	Spanish	733	9.8	56	100%	Grandparenting dimensions/Parenting Practices Interview (PPI)	Internalizing problem/the Strengths and Difficulties Questionnaire (SDQ)	NA	0.310	A
14. Y. Tan et al. (2023)	China	201	9.18	NA	100%	Psychological control or Behavioral control/self-compiled	Depression/Center for Epidemiological Studies-Depression (CES-D)	NA	0.105	A
15. L. F. Wang (2007)	China	359	6.17	NA	NA	Grandparenting dimensions/Child Rearing Practices Q-Sort (CRP-Q)	Mental health/Self-administered Early Childhood Mental Health Status Questionnaire	0.372	–0.539	A
16. Wu (2016)	China	211	NA	58	87%	Grandparenting dimensions/Classification Cards for Parenting Style Q (CRPR) Bloke	Mental health/Infant-Toddler Social and Emotional Assessment (ITSEA)	–0.010	0.180	B
17. Xue (2013)	China	127	NA	NA	NA	Grandparenting dimensions/Adapted from PBR	Autonomy/The Preschool Behavior Q-sort	0.090	–0.020	B
							Dependency/The Preschool Behavior Q-sort	0.050	–0.070	
18. Yan (2018)	China	853	9.98	NA	100%	Grandparenting dimensions/Adapted from EMBU	Depression/CBCL	–0.175	0.024	B
							Compulsive/CBCL	–0.139	–0.012	
19. Yang and Liu (2020)	China	1360	13.15	NA	NA	Grandparenting dimensions/Adapted from s-EMBU	Depression/The Birleson Depression Self-Rating Scale (DSRS)	–0.330	0.065	A
20. W. Y. Zhang (2021)	China	543	NA	NA	78.45%	Grandparenting dimensions/Adapted from EMBU	Depression/Middle School Student Mental Health Scale (MSSMHS)	–0.225	0.194	B
							Anxiety/MSSMHS	–0.170	0.179	
							Compulsive/MSSMHS	–0.080	0.208	
							Paranoia/MSSMHS	–0.180	0.228	
							Hostile/MSSMHS	–0.160	0.207	

Note: NA = not applicable.

## Data Availability

The data supporting the conclusions of this article will be made available by the authors upon request.
